# Adalimumab-Induced Thrombocytopenia: A Case Report of Drug-Induced Thrombocytopenia

**DOI:** 10.1155/crh/4414589

**Published:** 2025-09-08

**Authors:** Shelby E. Jones, Joshua Sellers, Margaret C. Wheless, Danielle Fishman, Robert K. McKenzie

**Affiliations:** ^1^Department of Pharmacy, College of Pharmacy and Health Sciences, Belmont University, 1900 Belmont Blvd, Nashville 37212, Tennessee, USA; ^2^Department of Medicine, Vanderbilt University Medical Center, 1211 Medical Center Dr, Nashville 37232, Tennessee, USA; ^3^Division of Hematology and Oncology, Vanderbilt University Medical Center, 1211 Medical Center Dr, Nashville 37232, Tennessee, USA; ^4^Division of Hematology Oncology, Vanderbilt University Medical Center, 1211 Medical Center Dr, Nashville 37232, Tennessee, USA

**Keywords:** adalimumab, autoimmune, case report, thrombocytopenia, TNF-alpha

## Abstract

In this case report, we present a patient with severe thrombocytopenia induced by adalimumab after 4 weeks of treatment for rheumatoid arthritis. This case adds to present literature regarding antitumor necrosis factor-alpha (TNF-α)-induced thrombocytopenia and explores the use of thrombopoietin receptor agonist medications in refractory drug-induced immune thrombocytopenia (DITP). A 57-year-old female with a history of rheumatoid arthritis presented to the emergency department for progressive petechial rash 4 days after her third dose of adalimumab. It was determined that the patient had immune thrombocytopenia caused by adalimumab and was initially treated with steroids and intravenous immunoglobulin (IVIG). Platelets remained undetectable; hence, romiplostim was initiated, after which platelets trended up to normal levels a week later. Anti-TNF-α agents carry the risk of severe side effects, including thrombocytopenia, which should be monitored closely in the first few months of therapy. The treatment of DITP is challenging, especially when the causative agent has a prolonged half-life.

## 1. Background

Antitumor necrosis factor-α (TNF-α) drugs can be an effective treatment for rheumatoid arthritis and other autoimmune conditions, though they do carry the risk of rare but serious side effects such as thrombocytopenia. There are several previously reported cases of adalimumab-induced thrombocytopenia [1–5]. Besides discontinuation of the offending agent, treatment of DITP remains poorly understood. This case highlights the challenges of DITP when the causative agent has a prolonged half-life and explores the use of thrombopoietin receptor agonists for refractory patients.

## 2. Case Presentation

A 57-year-old female diagnosed with rheumatoid arthritis in 2018 was initially managed with steroids and methotrexate but switched to adalimumab in June of 2024 due to persistent symptoms (40 mg subcutaneously every 14 days; received a total of 3 doses at weeks 0, 2, and 4). The patient presented to the emergency department 4 days after her third dose of adalimumab with a progressive petechial rash beginning a few days prior to admission and one episode of hematochezia. Prior to presentation, the patient reported feeling poorly with increased fatigue, decreased appetite, and several episodes of epistaxis. Labs on admission showed platelet count less than 5000 μL. Outpatient labs showed platelets of 166,000/μL 7 days prior to starting adalimumab and 135,000 μL 2 weeks after initiation. Other labs on the presentation showed an immature platelet fraction of 11.2%, hemoglobin 14.9 g/dL, white blood cells (WBC) 2.7 μL, PT 14.6 s, INR 1.1, PTT 31 s, and fibrinogen 427 mg/dL. Of note, leukopenia was stable with WBC in 2014 and 2020 of 2.8 μL and 3.1 μL, respectively. A peripheral flow and broad infectious workup were negative. Imaging did not reveal any cirrhosis, lymphadenopathy, or splenomegaly. Peripheral blood smear showed normocytic normochromic red blood cells and decreased WBC with normal morphology and neutrophil predominance. Rare platelets were seen with even fewer giant platelets; no platelet clumping to suggest pseudothrombocytopenia.

Given the patient's presentation and history of an autoimmune disease, dexamethasone 40 mg daily was given on hospital days 2 through 5 as standard immune thrombocytopenia (ITP) treatment. Prednisone 1 mg/kg was started on day 6 and continued until discharge due to continued thrombocytopenia. Intravenous immunoglobulin (IVIG) 1 g/kg was given on hospital days 2 and 3 and again on days 5 and 6. Despite these therapies, platelets remained undetectable through hospital day 7 without any major bleeding events. A bone marrow biopsy was performed on day 5 of treatment to rule out other causes of thrombocytopenia and showed normocellular marrow with trilineage hematopoiesis and no increase in blasts. Romiplostim 2 mcg/kg was given on hospital day 6 and again on day 9, given the prolonged duration of severe thrombocytopenia. Platelets were finally detectable on hospital day 8 and continued to trend up until discharge on day 13 to a value of 151,000 μL. Prior to discharge, the patient was given an additional dose of romiplostim.

## 3. Discussion and Conclusion

Thrombocytopenia is a well-established adverse effect of anti-TNF-α drugs, but there have only been three previously reported cases of adalimumab-induced thrombocytopenia [1–3].

In one case, a 73-year-old male with Crohn's disease was treated with infliximab and mesalazine. The patient was changed to adalimumab after the disease flared on infliximab. Shortly after starting adalimumab, the patient experienced epistaxis, cutaneous hematomas, and routine lab monitoring after 3 doses revealed thrombocytopenia with platelets of 24,000 μL. The authors of this report performed autoantibody testing for platelet antigens and found the presence of glycoprotein IIb/IIIa (GPIIb/IIIa) and glycoprotein V (GV) platelet receptor autoantibodies. Platelet counts started to recover 6 days after the last dose of adalimumab and fully recovered after 4 weeks [1].

In another case, a 71-year-old female was treated with azathioprine for Crohn's disease diagnosed in 1978. Adalimumab was added in October 2010 in addition to long-standing azathioprine. By January of 2011, the patient's routine labs revealed a platelet count of 44,000 μL. All other labs, imaging, and a bone marrow biopsy were normal. The patient was asymptomatic. A few days later the patient developed pneumonia and was admitted to the intensive care unit. Adalimumab and azathioprine were held during this admission, and platelets recovered to 113,000 μL. At discharge, azathioprine was held, and adalimumab was restarted. Three months after discharge platelets had again fallen to 25,000 μL and the patient was given IVIG and steroids with little response. Eventually the patient was changed to ustekinumab with stable platelets of 89,000 μL [2].

Lastly, a 42-year-old female with Crohn's disease developed thrombocytopenia with a platelet nadir of 44,000 μL after 6 doses of infliximab, another anti-TNF-α medication. Other causes of thrombocytopenia were ruled out. Bone marrow biopsy showed normal trilineage hematopoiesis with increased megakaryocytes. Further testing revealed platelet-associated IgG on the platelet surface via an unspecified platelet antibody test. The patient was then treated with oral prednisone for her Crohn's disease, and platelets recovered to normal levels after 5 months. The patient was then given a single dose of adalimumab, and platelet levels fell to less than 50,000 μL one week later [3].

This report adds to the growing body of evidence for adalimumab-induced thrombocytopenia and highlights the need for routine monitoring of platelets for the first few months of therapy. Of the now four cases of adalimumab-induced thrombocytopenia, all have shown a decrease in platelets within one to 3 months [1–3]. The manufacturer for adalimumab recommends baseline labs prior to starting adalimumab with repeat labs at 4 and 12 months [6]. We propose that a sooner platelet count check should be performed to detect thrombocytopenia and thus initiate earlier intervention.

Drug-induced thrombocytopenia is common and has many proposed mechanisms including immune and nonimmune pathways. The presence of antibodies toward GPIIb/IIIa, Ib/IX, and more recently GV has been present in many cases of suspected DITP, but little is known about how these antibodies are formed [7, 8]. Regardless of how antibodies are formed, an attack of glycoproteins in the cell membrane of platelets can lead to destruction of platelets. This is likely the case for adalimumab, highlighted by the reports presented by Salar et al., showing the presence of platelet autoantibodies from an unspecified test, and Boiten et al. who found GPIIb/IIIa and GV platelet autoantibodies via direct monoclonal antibody-specific immobilization of platelet antigens [1, 3].

There are well-established guidelines for the treatment of ITP, but the treatment of DITP, other than discontinuation of the offending drug, is less understood. This is especially true for adalimumab and drugs with longer half-lives. Usually, platelets will recover within 4-5 half-lives after discontinuation of the offending drug in DITP. Platelet transfusions are typically not helpful if the drug is still in the body since the change in platelet surface makes an easy target for antibodies. The efficacy of IVIG has only been shown in one case report [9]. Adalimumab has a half-life of 7 days, which is concerning for cases such as this where the degree of thrombocytopenia is severe. Romiplostim, along with eltrombopag, quickly stimulates the proliferation of megakaryocytes into mature platelets [10, 11]. The significant dollar cost of these medications usually limits their inpatient use, but our case highlights their potential importance for refractory thrombocytopenia in DITP with drugs with prolonged half-lives. The available biopsy reports in adalimumab-induced thrombocytopenia have shown either normal or elevated megakaryocytes, making the thrombopoietin receptor agonists a logical option for other cases with severe DITP in the setting of adalimumab.

DITP is a recognized complication of adalimumab and other TNF-α inhibitors that should be monitored closely, particularly in the first few months with adalimumab. The use of thrombopoietin receptor agonists can be considered for refractory cases of DITP when the causative agent has a prolonged half-life.

## Data Availability

The data supporting this case study are not publicly available but are available on request due to privacy of the patient.

## Nomenclature

DITP: Drug-induced thrombocytopenia
dL: Deciliter
GPIIb/IIIa: Glycoprotein IIb/IIIa
GV: Glycoprotein V
Ig-G: Immunoglobulin G
INR: International normalized ratio
ITP: Immune thrombocytopenia
IVIG: Intravenous immunoglobulin
Mcg: Microgram
μL: Microliter
PT: Prothrombin time
PTT: Partial thromboplastin time
TNF-α: Tumor necrosis factor alpha
WBC: White blood cells
